# Seroprevalence of Epstein–Barr virus infection in children during the COVID-19 pandemic in Zhejiang, China

**DOI:** 10.3389/fped.2023.1064330

**Published:** 2023-02-09

**Authors:** Fengqing Cai, Hui Gao, Qing Ye

**Affiliations:** Department of Clinical Laboratory, The Children’s Hospital, Zhejiang University School of Medicine, National Clinical Research Center for Child Health, National Children’s Regional Medical Center, Hangzhou, China

**Keywords:** Epstein–Barr virus, COVID-19 pandemic, children, seroprevalence, infections

## Abstract

**Aim:**

We aimed to investigate the seroprevalence of Epstein–Barr virus (EBV) infection in children before and during the COVID-19 pandemic.

**Methods:**

All children admitted to the Children's Hospital Affiliated to Zhejiang University from January 2019 to December 2021 with suspected EBV-associated disease and EBV antibodies were detected by a two-step indirect method of chemiluminescence technology. A total of 44,943 children were enrolled in this study. The seroprevalence of EBV infections was compared from January 2019 to December 2021.

**Results:**

The total seropositive rate of EBV infections was 61.02% between January 2019 and December 2021, and the seropositive trend decreased year by year. The total number of seropositive EBV infections in 2020 was reduced by 30% compared to that in 2019. In particular, nearly 30% and 50% reductions in the number of acute EBV infections and EBV reactivations or late primary infections from 2019 to 2020 were found, respectively. The number of acute EBV infections in children aged 1–3 years and EBV reactivation or late primary infection in children aged 6–9 years in 2020 sharply dropped by approximately 40% and 64% compared to that in 2019.

**Conclusions:**

Our study further demonstrated that the prevention and control measures for COVID-19 in China had a certain effect on containing acute EBV infections and EBV reactivations or late primary infections.

## Introduction

Epstein‒Barr virus (EBV), belonging to human herpesvirus 4 (HHV4), is an important pathogen of infection in children. EBV can not only cause acute or transient infection but is also associated with infectious mononucleosis (IM), hemophagocytic lymph histiocytosis (HLH), lymphoma, etc. (1–5). EBV infection included two states: proliferative infection (or acute infection) and latent infection. EBV infected human lymphocytes and epithelial cells. After initial infection, EBV can be latent in human upper respiratory epithelial cells or lymphoid tissues for a long time. Latent infection and long-term carrier status are important characteristics of EBV infection (6, 7). Serological tests for EBV antibodies are frequently applied to explain infection status (8). Epstein‒Barr virus capsid antigen (VCA)-IgG, VCA-IgM and EBV nuclear antigen (EBNA)-IgG were the three antibodies specific for EBV. The different antibody patterns were used to distinguish acute from past EBV infection (9).

EBV infection is prevalent worldwide, and approximately 90% of adults show serological antibody positivity throughout the world (6). Primary EBV infection in United States children develops in late adolescence (10); furthermore, primary EBV infection develops at an early age in developing areas (11). The oral route is the primary route of EBV transmission (12). Accordingly, the seroprevalence in children is inconsistent according to socioeconomic status, sanitary conditions and educational level (10). A study on the global burden of EBV-related cancer estimated that EBV-related cases accounted for 239,700–357,900 new cases and 137,900–208,700 deaths in 2020 (1). Recently, some studies found that COVID-19 patients were seropositive for EBV VCA-IgM antibody, and EBV reactivation may be associated with the severity of COVID-19 (13–16). Since the outbreak of COVID-19, many preventive and control measures in China have been implemented to curb the spread of severe acute respiratory syndrome coronavirus (SARS-CoV-2) (17–20) and improve the infection of other pathogens, such as common children's respiratory viruses, *Mycoplasma pneumoniae* and *Chlamydia pneumoniae* (21, 22). These measures refer to nonpharmaceutical interventions (23), consisting of containment and suppression. Personal protective measures include wearing masks, practicing hand hygiene, and maintaining social distance (24). Meanwhile, with the improvement of economic and health standards in our country, the seroprevalence of EBV infection needs to be determined.

The goal of this study was to compare the seroprevalence of EBV infection in children before and during the COVID-19 pandemic.

## Methods

### Study subjects

All children admitted to the Children's Hospital Affiliated to Zhejiang University from January 2019 to December 2021, including outpatients and inpatients who were subjected to EBV antibody assays, were included in this study. The inclusion criteria were ① patients who were suspected to have EBV-associated diseases (6) and ② patients under 18 years old. The exclusion criteria were ① patients with repeated tests in one age group and ② patients infected with COVID-19. Finally, a total of 44,943 children were enrolled in this study from January 2019 to December 2021. Demographic data such as age and sex were obtained from electronic medical records. All the children were divided into six age groups: under 0–6 months (0–6 m), 6–12 months (6–12 m), 1–3 years (1–3 y), 3–6 years (3–6 y), 6–9 years (6–9 y) and >9 years (>9 y). The positive rate of EBV antibody was also compared by month.

### Detection of EBV antibody

After admission, blood was collected with a heparin anticoagulant tube and then centrifuged for 5 min at 2,500 r/min. Centrifuged serum was used for detection. VCA-IgM, VCA-IgG and EBNA-IgG antibodies were detected by a two-step indirect method of chemiluminescence technology (iFlash3000, YHLO, Shenzhen, China) following the manufacturer's instructions. First, the serum was reacted with EBV antigen coated with magnetic particles to form an antigen-antibody complex. Unbound impurities were washed away. Second, acridine-labeled mouse anti-human IgM/IgG was added to the tube to form an antigen-antibody-double antibody complex. Unbound impurities were washed away again. Finally, preexcitation solution and excitation solution were added to the reaction tube, and the relative luminous intensity (RLU) of the mixture was detected by the optical system of the tester. The concentration of EBV antibody was calculated according to RLU. When the concentration of VCA-IgM was <20 U/ml, the IgM antibody was negative. When the concentration of VCA-IgM was between 20 and 40 U/ml, the result needed to be rechecked or comprehensively judged. When the concentration of VCA-IgM was ≥40 U/ml, the VCA-IgM antibody was positive. When the concentration of VCA-IgG/EBNA-IgG was <20 U/ml, the IgG antibody was negative. When the concentration of VCA-IgG/EBNA-IgG was ≥20 U/ml, the IgG antibody was positive.

The EBV infection status of patients was classified according to different antibody patterns (9) (Table 1).

**Table 1:** Common EBV antibody patterns.

Patterns	VCA-IgM	VCA-IgG	EBNA-IgG	EBV infection status
A	+	–	–	Acute EBV infection
B	+	+	–	Acute EBV infection
C	–	+	–	Past EBV infection
D	–	+	+	Past EBV infection
E	–	–	+	Past EBV infection
F	+	+	+	EBV reactivation or late primary infection
G	–	–	–	seronegative

### Statistical analysis

All the data were analyzed using SPSS version 26.0 software (IBM Corp., Armonk, N.Y., United States). The figures were generated using GraphPad Prism (version 9.0, La Jolla, CA, United States). Categorical variables were analyzed using the *χ*^2^ test or Fisher's exact test. A *P* value <0.05 was considered statistically significant.

## Results

### Patient characteristics

From January 2019 to December 2021, a total of 44,943 children were enrolled in this study, including 16,462 cases in 2019, 12,582 cases in 2020 and 15,899 cases in 2021. The number of patients in 2020 decreased by nearly 4,000 cases compared with that in 2019 and 2021. The male to female ratio was 1.35:1 (25,804:19,139). There was no significant difference in sex between patients among the three years of 2019–2021. The detection numbers and rates of EBV antibodies in children at 0–6 months (913/12,582, 7.26%), 1–3 years (3,342/12,582, 26.56%) and 6–9 years (1,574/12,582, 12.51%) in 2020 were less than those in 2019 (1,342/16,462, 8.15%; 4,766/16,462, 28.95; 2,359/16,462, 14.33%). However, most patients were still concentrated in 1–6 years (26,591/44,943, 59.17%) (Table 2).

**Table 2:** Patient characteristics and detection of EBV antibodies between January 2019 and December 2021.

	All (*n* = 44,943)	2019 (*n* = 16,462)	2020 (*n* = 12,582)	2021 (*n* = 15,899)	*χ^2^* value	*P* value
**Characteristics, *n* (%)**						
Age						
0–6 month	3,241 (7.21)	1,342 (8.15)	913 (7.26)	986 (6.20)	46.035	<0.001
6–12 month	3,141 (6.99)	1,099 (6.68)	1,075 (8.54)	967 (6.08)	63.775	<0.001
1–3 years	12,529 (27.88)	4,766 (28.95)	3,342 (26.56)	4,421 (27.81)	203.234	<0.001
3–6 years	14,062 (31.29)	4,745 (28.82)	3,827 (30.42)	5,490 (34.53)	369.515	<0.001
6–9 years	5,886 (13.10)	2,359 (14.33)	1,574 (12.51)	1,953 (12.28)	47.439	<0.001
>9 years	6,084 (13.54)	2,151 (13.07)	1,851 (14.71)	2,082 (13.10)	3.093	0.213
Sex						
Male	25,804 (57.41)	9,480 (57.59)	7,159 (56.90)	9,165 (57.65)	1.915	0.384
Female	19,139 (42.59)	6,982 (42.41)	5,423 (43.10)	6,734 (42.35)		
**Detection of EBV antibodies, *n* (%)**						
VCA-IgM (+)	7,016 (15.61)	2,795 (16.98)	1,822 (14.48)	2,399 (15.09)	38.852	<0.001
VCA-IgG (+)	21,058 (46.85)	8,138 (49.44)	5,798 (46.08)	7,122 (44.80)	70.466	<0.001
EBNA-IgG (+)	21,415 (47.65)	8,424 (51.17)	5,883 (46.76)	7,108 (44.71)	116.268	<0.001
**EBV antibody patterns, *n* (%)**						
A	4,315 (9.60)	1,645 (9.99)	1,135 (9.02)	1,535 (9.65)	7.843	0.020
B	1,156 (2.57)	396 (2.42)	312 (2.48)	448 (2.82)	6.080	0.048
C	536 (1.19)	191 (1.16)	170 (1.35)	175 (1.10)	3.969	0.137
D	17,893 (39.81)	6,850 (41.61)	4,952 (39.36)	6,091 (38.31)	38.274	<0.001
E	1,977 (4.40)	820 (4.98)	556 (4.42)	601 (3.78)	27.760	<0.001
F	1,545 (3.44)	754 (4.58)	375 (2.98)	416 (2.62)	104.960	<0.001
Total	27,422 (61.02)	10,656 (64.73)	7,500 (59.61)	9,266 (58.28)	156.000	<0.001

### Changes in overall seropositive rates of EBV infections

Of the 44,943 children, the seropositive rates of EBV-specific antibodies, including VCA-IgM, VCA-IgG and EBNA-IgG, were 15.61%, 46.85% and 47.65%, respectively. In 2019, 2,795 patients were seropositive for VCA-IgM, 8,138 were seropositive for VCA-IgG, and 8,424 patients were seropositive for EBNA-IgG. In 2020, the positive numbers of VCA-IgM, VCA-IgG and EBNA-IgG markedly decreased to 1,822, 5,798 and 5,883, respectively. The number of VCA-IgM-positive patients in 2020 was reduced by approximately 35% compared with that in 2019 (1,822 vs. 2,795, *P *< 0.001). However, the positive numbers of three EBV-specific antibodies increased again in 2021 but were still lower than those in 2019 (VCA-IgM: 2,399 vs. 2,795, VCA-IgG: 7,122 vs. 8,138, EBNA-IgG: 7,108 vs. 8,424, *P *< 0.001) (Table 2).

We analyzed common EBV antibody patterns to explain the different phases of EBV infection. As shown in Table 2, the total seropositive rate of EBV infection was 61.02% between January 2019 and December 2021, and the seropositive trend decreased yearly (64.73% in 2019, 59.61% in 2020 and 58.28% in 2021). The total number of seropositive EBV infections in 2020 was reduced by approximately 30% compared to that in 2019 (7,500 vs. 10,656, *P *< 0.001).

Patterns D and E, which were the predominant status of typical characteristics of past EBV infection, accounted for 39.81% and 4.40% of all the enrolled children, respectively. Patterns A and B were typical statuses of acute EBV infection, accounting for 9.60% and 2.57% of all children, respectively. Pattern F indicated EBV reactivation or late primary infection. Interestingly, there was a nearly 30% reduction in the number of acute infections (2,041 vs. 1,447, *P *= 0.02) and a nearly 50% reduction in the number of EBV reactivations or late primary infections (754 vs. 375, *P *< 0.001) from 2019 to 2020 (Table 2).

### Obvious age distribution trends of EBV infections

**Figure 1:**
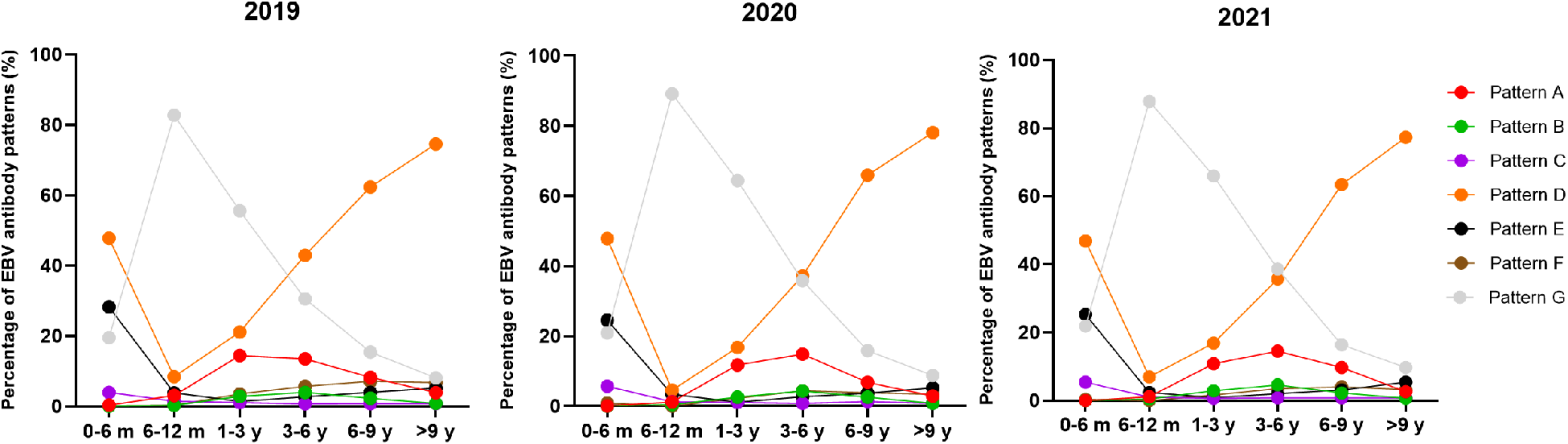
The percentage of EBV antibody patterns based on age between January 2019 and December 2021.

The overall positive rate of EBV antibody patterns based on age is shown in Table 3. The overall seropositive rates reached 79.36% at ages 0–6 months, then declined to 13.50% at ages 6–12 months, and subsequently increased gradually in the older age groups to a peak of 91.14% at ages >9 years. The trends in seropositive rates of age distribution between 2019 and 2021 were similar (Figure 1). However, the seropositive rates of children aged 6 months-6 years in 2020 were lower than those in 2019 (6–12 months: 10.88% vs. 17.29%, *P *< 0.001, 1–3 years: 35.61 vs. 44.40%, *P *< 0.001, 3–6 years: 64.20% vs. 69.48%, *P *= 0.002). The positive numbers peaked in the 3–6 years group, with peaks of 3,297 cases in 2019, 2,457 cases in 2020 and 3,366 cases in 2021 (Table 3).

**Table 3:** The overall positive rate of EBV antibody patterns based on age between January 2019 and December 2021.

Age	All	2019	2020	2021	*χ^2^* value	*P* value
0–6 months	2,572/3,241 (79.36)	1,080/1,342 (80.48)	722/913 (79.01)	770/986 (78.09)	44.226	<0.001
6–12 months	424/3,141 (13.50)	190/1,099 (17.29)	117/1,075 (10.88)	117/967 (12.20)	15.176	0.001
1–3 years	4,808/12,529 (38.37%)	2,116/4,766 (44.40)	1,190/3,342 (35.61)	1,502/4,421 (33.97)	126.376	<0.001
3–6 years	9,120/14,062 (64.86)	3,297/4,745 (69.48)	2,457/3,827 (64.20)	3,366/5,490 (61.31)	12.849	0.002
6–9 years	4,953/5,886 (84.15)	1,996/2,359 (84.61)	1,325/1,574 (84.18)	1,632/1,953 (83.56)	32.810	<0.001
>9 years	5,545/6,084 (91.14)	1,977/2,151 (91.91)	1,689/1,851 (91.25)	1,879/2,082 (90.25)	19.331	<0.001

Data are expressed as the positive number/the total number per age group (%).

**Figure 2:**
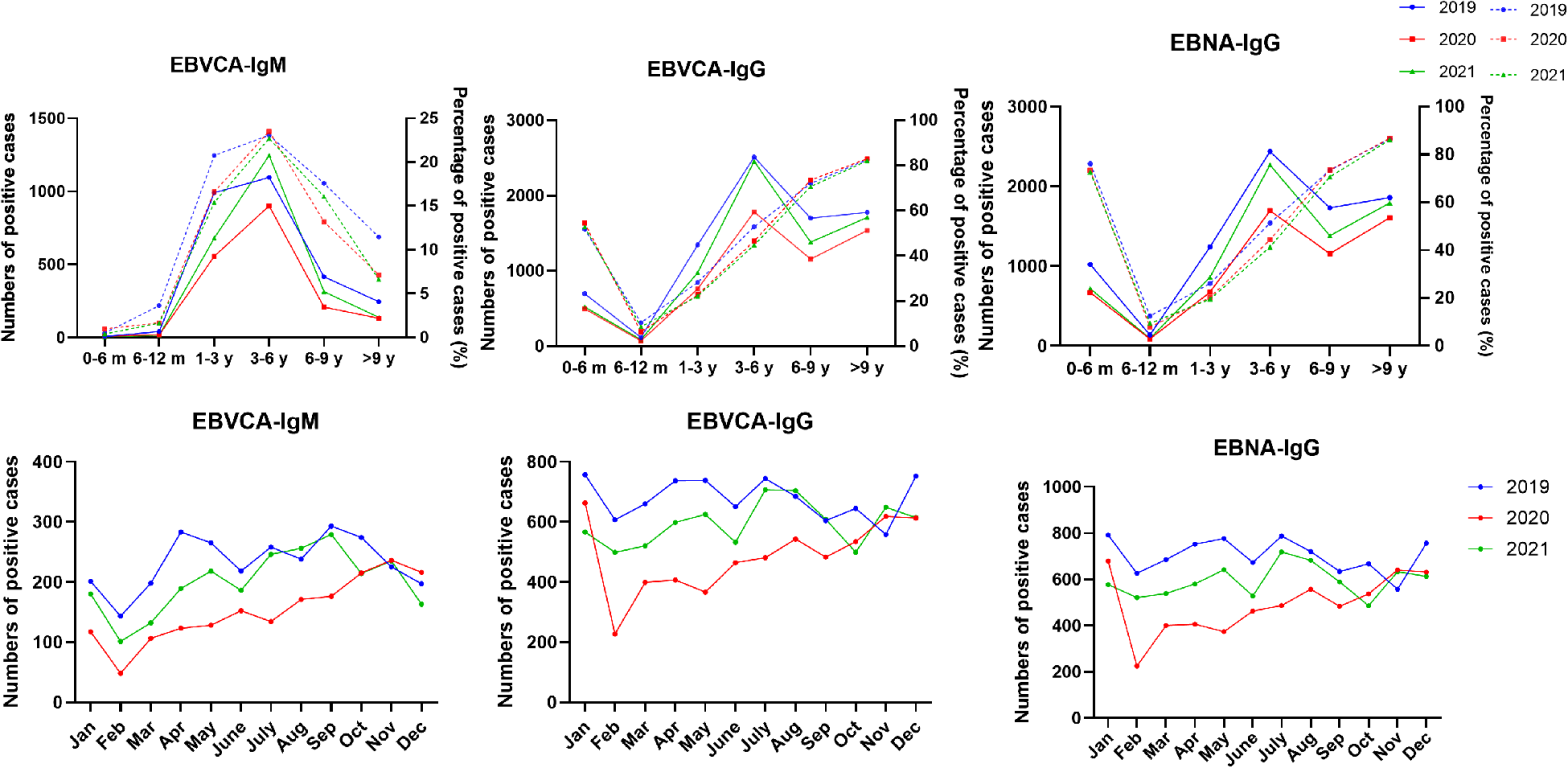
The distribution of EBV antibodies based on age and month between January 2019 and December 2021. The vertical axis on the left and solid lines represent the number of EBV antibody-positive cases. The vertical axis on the right and dashed lines represent the percentage of EBV antibody-positive cases.

As shown in Figure 2, the seropositive rates of both VCA-IgG and EBNA-IgG at 0–6 months of age were nearly 60% and 80%, respectively. They dropped to the lowest point in children aged 6–12 months and then gradually increased to more than 80% with age. The seropositive rates of VCA-IgM increased from 0.5% at 0–6 months of age to a peak of nearly 23% at 3–6 years of age and then dropped with age. The changes in seropositive rates of EBV antibodies based on age showed similar trends between 2019 and 2021. In contrast, the positive numbers of VCA-IgM, VCA-IgG and EBNA-IgG at age >1 year in 2020 were significantly lower than those in 2019 and 2021.

Simultaneously, different EBV antibody patterns based on age during 2019–2021 are shown in Table 4 and Supplementary Figure S1. Patterns D and E, showing EBV past infection, were mainly over 3 years old. The percentages of patterns D and E increased with age. In particular, pattern D was most prevalent in children aged >3 years. The children in patterns A and B, indicating EBV acute infection, were mainly concentrated in 1–6 years. From 2019 to 2021, acute EBV infection in children aged 1–3 years and 3–6 years was significantly higher than that in children in the other groups. In 2020, the number of acute EBV infections in children aged 1–3 years sharply dropped by 40% compared to that in 2019 (pattern A: 393 vs. 688, pattern B: 86 vs. 135). The change in the incidence of pattern F, which may indicate EBV reactivation or late primary infection, was chiefly observed in children aged >1 year from 2019 to 2021. In particular, there was an approximately 64% reduction in the positive number of children aged 6–9 years with EBV reactivation or late primary infection from 2019 to 2020 (pattern F: 167 vs. 60).

**Table 4:** Detection of different EBV antibody patterns based on age between January 2019 and December 2021.

EBV antibody patterns	Year	0–6 months	6–12 months	1–3 years	3–6 years	6–9 years	>9 years	*χ^2^* value	*P* value
**A**	**2019**	5 (0.37)	34 (3.09)	688 (14.44)	641 (13.51)	194 (8.22)	83 (3.86)	1,776.4	<0.001
	**2020**	1 (0.11)	13 (1.21)	393 (11.76)	568 (14.84)	107 (6.80)	53 (2.86)	1,485.5	<0.001
	**2021**	0 (0.00)	11 (1.14)	481 (10.88)	798 (14.54)	190 (9.73)	55 (2.64)	2,044.7	<0.001
**B**	**2019**	0 (0.00)	3 (0.27)	135 (2.83)	186 (3.92)	54 (2.29)	18 (0.84)	455.3	<0.001
	**2020**	0 (0.00)	5 (0.47)	86 (2.57)	164 (4.29)	41 (2.60)	16 (0.86)	386.8	<0.001
	**2021**	0 (0.00)	5 (0.52)	128 (2.90)	255 (4.64)	45 (2.30)	15 (0.72)	675.9	<0.001
**C**	**2019**	53 (3.95)	16 (1.46)	52 (1.09)	35 (0.74)	18 (0.76)	17 (0.79)	48.1	<0.001
	**2020**	52 (5.70)	14 (1.30)	37 (1.11)	31 (0.81)	21 (1.33)	15 (0.81)	38.2	<0.001
	**2021**	54 (5.48)	10 (1.03)	31 (0.70)	45 (0.82)	17 (0.87)	18 (0.86)	51.9	<0.001
**D**	**2019**	641 (47.76)	92 (8.37)	1,007 (21.13)	2,037 (42.93)	1,470 (62.31)	1,603 (74.52)	2,346.2	<0.001
	**2020**	437 (47.86)	49 (4.56)	560 (16.76)	1,423 (37.18)	1,038 (65.95)	1,445 (78.07)	2,088.1	<0.001
	**2021**	462 (46.86)	68 (7.03)	749 (16.94)	1,963 (35.76)	1,239 (63.44)	1,610 (77.33)	2,710.9	<0.001
**E**	**2019**	379 (28.24)	42 (3.82)	67 (1.41)	128 (2.70)	93 (3.94)	111 (5.16)	554.7	<0.001
	**2020**	224 (24.53)	36 (3.35)	38 (1.14)	103 (2.69)	58 (3.68)	97 (5.24)	269.3	<0.001
	**2021**	250 (25.35)	23 (2.38)	41 (0.93)	112 (2.04)	62 (3.17)	113 (5.43)	338.2	<0.001
**F**	**2019**	2 (0.15)	3 (0.27)	167 (3.50)	270 (5.69)	167 (7.08)	145 (6.74)	440.7	<0.001
	**2020**	8 (0.88)	0 (0.00)	76 (2.27)	168 (4.39)	60 (3.81)	63 (3.40)	292.6	<0.001
	**2021**	4 (0.41)	0 (0.00)	72 (1.63)	193 (3.52)	79 (4.05)	68 (3.27)	354.5	<0.001

Data are expressed as the positive number (%: positive number of total numbers per age stage).

### Weak seasonal distribution variation in EBV infections

Weak seasonal variation was observed in the overall positive rates of the EBV antibody pattern during 2019–2021. The positive numbers of EBV antibody patterns from February to October 2020 were lower than those in 2019 and 2021. (Supplementary Table S1) Similarly, the seasonal distribution trends of VCA-IgG and EBNA-IgG were consistent in 2019 and 2021 but not in 2020. From February to October 2020, the numbers of EBVCA-IgM-, EBV-IgG- and EBNA-IgG-positive cases decreased obviously compared with those in the other two years (Figure 2).

**Figure 3:**
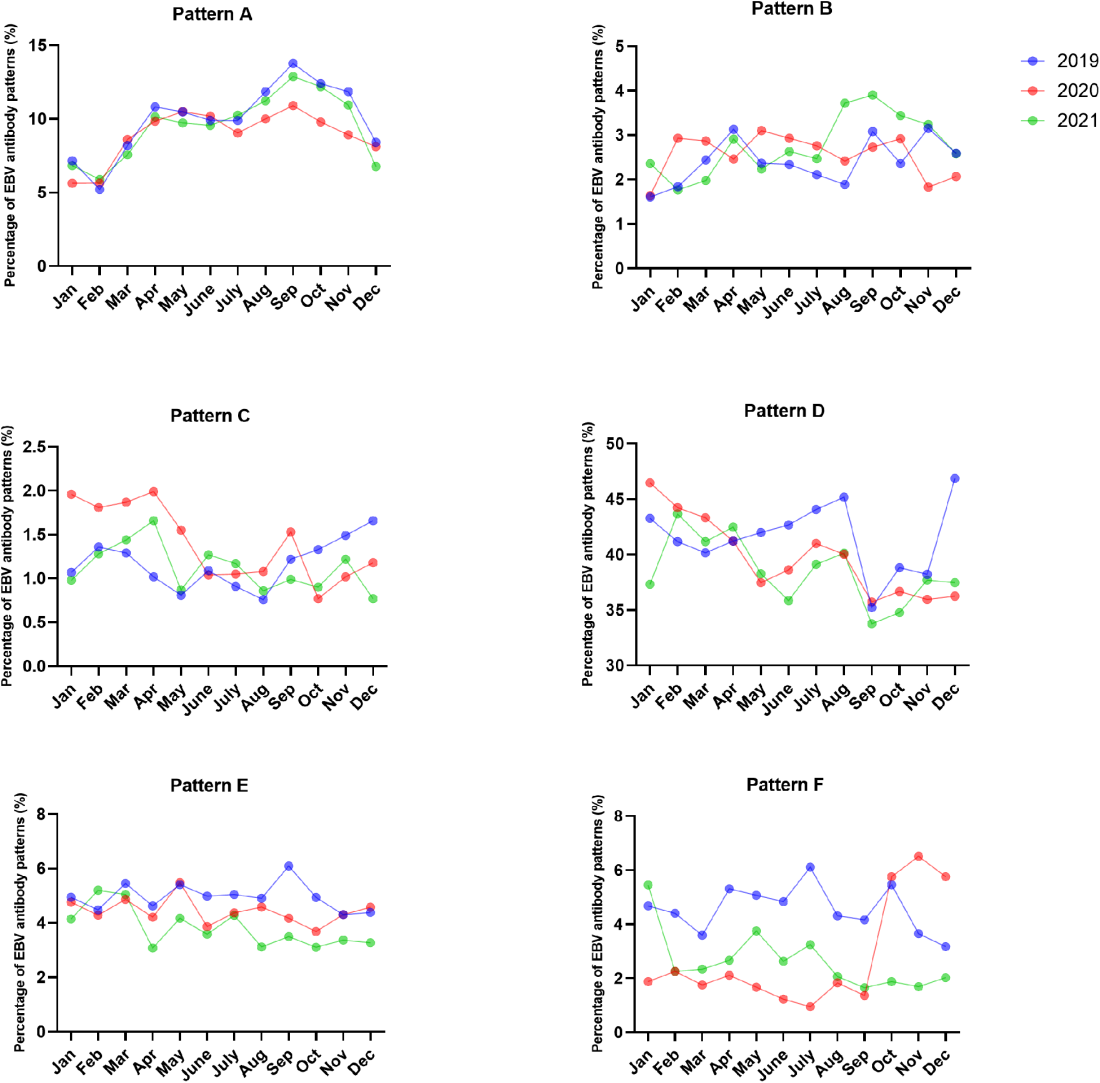
The percentages of EBV antibody patterns based on months between January 2019 and December 2021.

**Figure 4:**
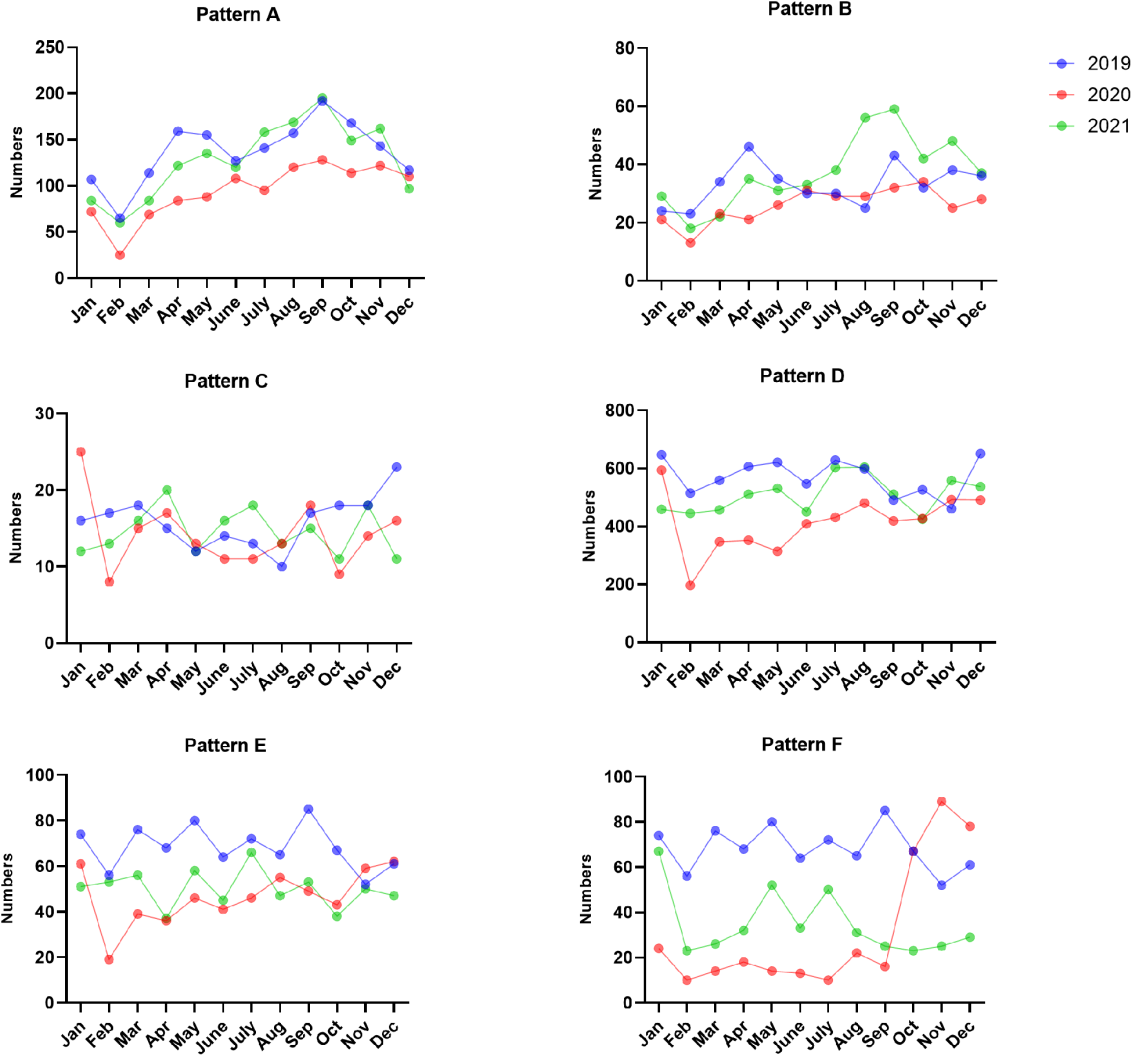
The numbers of EBV antibody patterns based on months between January 2019 and December 2021.

The most common EBV infection status throughout the year was pattern D, accounting for 35.22%–46.87% in 2019, 35.72%–46.48% in 2020 and 33.75%–43.67% in 2021. Interestingly, the percentage of pattern A increased from August to November and peaked in September (13.77% in 2019, 10.91% in 2020 and 12.88% in 2021). The numbers of acute EBV infections and EBV reactivations or late primary infections from January to September 2020 significantly decreased compared to those in 2019 (Figures 3, 4 and Supplementary Table S2).

## Discussion

EBV is prevalent throughout the world. In recent decades, some studies have reported epidemiological changes in EBV infections. The prevalence of EBV in Japan decreased to 59% for the years 1995–1999 from >80% before early 1990 (25). EBV infection in France has decreased during the last 15 years, and the seronegative rates increased from nearly 50% during 2001–2005 to >60% during the 2011–2015 period in patients aged <10 years old (26). A previous study in other regions of China found that the positive rates of EBV infection reached 78.0% in children aged 0–16 years (27). Similarly, a retrospective study showed that the prevalence of EBV infection in North and South China was 80.78% and 79.38% in children aged 0–10 years, respectively (28). Recently, Jeffrey et al. found an association between long reactivation of EBV and COVID symptoms in 66.7% of COVID-19 patients (29). In our study, the total seropositive rate of EBV infection was 61.02% among 44,943 children aged 0–18 years between January 2019 and December 2021. The lower seropositive rate of EBV infection in our study may be due to differences in the study population; children ranging from 1 year to 6 years accounted for 59.17%, and the total number of enrolled patients was tremendous. This finding indicated that the EBV infection rate of young children has decreased.

VCA-IgG appears after the initial EBV infection and persists throughout life. EBNA-IgG appears three months after the initial EBV infection and persists throughout life (12, 30). Pattern D and pattern E represented past EBV infection, accounting for 44.21%. However, other investigations showed that the positivity rate of VCA-IgG was 74.6% in children aged 0–16 years in Suzhou (27), 77.8% in Guangzhou and 77.4% in Beijing (28). Similarly, the positivity rate of EBNA-IgG in our study was lower than that in Guangzhou (75.53%) and Beijing (76.26%). This finding indicated that EBV infection had improved in the difficult region and study population.

EBV is the primary cause of infectious mononucleosis and is relevant to several malignancies, including Burkitt lymphoma, Hodgkin lymphoma, multiple sclerosis and hemophagocytic syndrome. At present, there is no available vaccine against EBV. A previous study showed that monomeric EBV gp350 can reduce the incidence of infectious mononucleosis in a phase 2 trial but cannot reduce the rate of EBV infection (31). We were surprised to find that the prevalence of VCA-IgM in 2020, which was judged as a parameter of acute EBV infection, was less than that in 2019 and 2021. Equally, pattern A, a common EBV antibody pattern, is a typical serological status of acute EBV infection (9). VCA-IgM appears after the initial EBV infection, then rises rapidly during acute infection and disappears after nearly four weeks (8). Compared to those in 2019 and 2021, the numbers of acute infections decreased obviously in 2020. The decrease in acute EBV infection showed the significant additional effect of these preventive and control measures to contain COVID-19 in China, which was consistent with our previous results (21). EBV generally spreads between young children through salivary contact and can cause clinical illness once immune function declines (30). To some extent, personal protection measures, consisting of wearing masks and washing hands frequently (32), can reduce virus transmission in saliva.

In our study, the EBV seropositivity rate, mainly for both VCA-IgG and EBNA-IgG, was higher in children aged 0–6 months than in children aged 6–12 months because maternal antibodies transfer through the placenta (33). After these two antibodies disappeared at the age of 6 months, they declined to the lowest level at 6–12 months of age and then increased gradually with age. This observation was consistent with a previous study (27, 28). The high seropositivity rate at age >6 years was chiefly due to rising VCA-IgG and EBNA-IgG, which was also similar to other obvious studies in the United States (10), Brazil (34) and Beijing (35). Instead, the seropositive rate of VCA-IgM in children aged >6 years decreased by degrees. This may be due to the growing immune system with age defending against the virus. The extremely low positive rates of VCA-IgM in children aged <1 year may be associated with maternal antibodies after birth (36). The changes in EBV antibody positivity based on age showed similar trends between 2019 and 2021. In 2020, the number of acute EBV infections in children aged 1–3 years sharply dropped by 40% compared to that in 2019. This may happen because children had relatively less contact with the saliva of other people infected with EBV after the COVID-19 pandemic.

Since Zhejiang Province promoted the 1-level emergency response on January 23, 2020, the number of seropositive cases decreased sharply in February 2020, and the positive numbers of EBV infections were lower than those in 2019 and 2021. The numbers of acute EBV infections and EBV reactivations or late primary infections from January to September 2020 significantly decreased compared to those in 2019. This finding further showed that the prevention and control measures in China had a certain effect on containing EBV infection. The incidence of acute EBV infection increased from August to November. This may be due to school openings during this period, as children had more frequent contact.

In summary, these nonpharmaceutical interventions against COVID-19 to contain the incidence of acute EBV infection to a certain extent may be attributed to some factors. First, face masks can effectively prevent the spread of EBV from infected people. Some studies found that the positive rates of various respiratory viruses, atypical pathogens and enteroviruses decreased during COVID-19 (21, 37, 38). Second, hand disinfection may eliminate EBV because almost all enveloped viruses can be killed by 75% alcohol.

Nevertheless, our study had some limitations. First, this was a single-center study, so case selection may be variant. This hospital is a comprehensive tertiary children's hospital in Zhejiang Province, China. The seropositive rate in our study may differ from that in other regions in China owing to economic levels and lifestyles. Second, our data only contained 3 years of medical records. This relatively short period may be incapable of observing obvious changes between years. Accordingly, further study may track trends in the epidemiological characteristics of EBV infection over a long period.

## Conclusion

The prevention and control measures for COVID-19 in China had a certain effect on containing acute EBV infections and EBV reactivations or late primary infections.

## Data Availability

The original contributions presented in the study are included in the article/**Supplementary Material**, further inquiries can be directed to the corresponding author.

## Supplementary material

Supplement Figure S1 The number of EBV antibody patterns based on age between January 2019 and December 2021.
