# Supplementary material for: Seroprevalence of Epstein–Barr virus infection in children during the COVID-19 pandemic in Zhejiang, China
Source: Front Pediatr. 2023 Feb 9;11:1064330. doi: 10.3389/fped.2023.1064330 (PMC9947643; doi:10.3389/fped.2023.1064330)
Supplement: Supplementary file 1 [file Table1.docx]

**Table S1. The overall positive rate of EBV antibody patterns based on month between January 2019 and December 2021**

| Month | **All** | **2019** | **2020** | **2021** | *χ^2^* value | *p* value |
| --- | --- | --- | --- | --- | --- | --- |
| **January** | 2437/4003 (60.88) | 938/1495 (62.74) | 797/1278 (62.36) | 702/1230 (57.07) | 54.285 | <0.001 |
| **February** | 1614/2713 (59.49) | 731/1251 (58.43) | 271/443 (61.17) | 612/1019 (60.06) | 112.434 | 0.001 |
| **March** | 2019/3303 (61.13) | 851/1392 (61.14) | 507/801 (63.30) | 661/1110 (59.55) | 28.028 | <0.001 |
| **April** | 2257/3527 (63.99) | 972/1470 (66.12) | 528/854 (61.83) | 757/1203 (62.93) | 47.122 | <0.001 |
| **May** | 2298/3704 (62.04) | 978/1479 (66.13) | 501/838 (59.79) | 819/1387 (59.05) | 56.487 | <0.001 |
| **June** | 2154/3596 (59.90) | 844/1282 (65.83) | 613/1059 (57.88) | 697/1255 (55.54) | 10.027 | <0.001 |
| **July** | 2526/4017 (62.88) | 971/1425 (68.14) | 622/1051 (59.18) | 933/1541 (60.55) | 15.107 | 0.001 |
| **August** | 2551/4028 (63.33) | 912/1324 (68.88) | 719/1199 (59.97) | 920/1505 (61.13) | 0.966 | 0.617 |
| **September** | 2406/4081 (58.96) | 886/1394 (63.56) | 662/1173 (56.44) | 858/1514 (56.67) | 0.295 | 0.863 |
| **October** | 2267/3741 (60.60) | 885/1355 (65.31) | 694/1164 (59.62) | 688/1222 (56.30) | 26.686 | <0.001 |
| **November** | 2418/4054 (59.64) | 756/1206 (62.69) | 801/1368 (58.55) | 861/1480 (58.18) | 44.139 | <0.001 |
| **December** | 2475/4176 (59.27) | 932/1389 (67.10) | 785/1354 (57.98) | 758/1433 (52.90) | 30.418 | <0.001 |

Data are expressed as the positive number/the total number per month (%).
