# Supplementary material for: Seroprevalence of Epstein–Barr virus infection in children during the COVID-19 pandemic in Zhejiang, China
Source: Front Pediatr. 2023 Feb 9;11:1064330. doi: 10.3389/fped.2023.1064330 (PMC9947643; doi:10.3389/fped.2023.1064330)
Supplement: Supplementary file 2 [file Table2.docx]

**Table S2. Detection of different EBV antibody patterns based on month** **between January 2019 and December 2021**

| **EBV antibody patterns** | **Year** | **January** | **February** | **March** | **April** | **May** | **June** | **July** | **August** | **September** | **October** | **November** | **December** | *χ^2^* value | *p* value |
| --- | --- | --- | --- | --- | --- | --- | --- | --- | --- | --- | --- | --- | --- | --- | --- |
| **A** | **2019** | 107 | 65 | 114 | 159 | 155 | 127 | 141 | 157 | 192 | 168 | 143 | 117 | 90.9 | <0.001 |
|  | **2020** | 72 | 25 | 69 | 84 | 88 | 108 | 95 | 120 | 128 | 114 | 122 | 110 | 100.9 | <0.001 |
|  | **2021** | 84 | 60 | 84 | 122 | 135 | 120 | 158 | 169 | 195 | 149 | 162 | 97 | 144.0 | <0.001 |
| **B** | **2019** | 24 | 23 | 34 | 46 | 35 | 30 | 30 | 25 | 43 | 32 | 38 | 36 | 17.4 | 0.097 |
|  | **2020** | 21 | 13 | 23 | 21 | 26 | 31 | 29 | 29 | 32 | 34 | 25 | 28 | 14.5 | 0.207 |
|  | **2021** | 29 | 18 | 22 | 35 | 31 | 33 | 38 | 56 | 59 | 42 | 48 | 37 | 45.6 | <0.001 |
| **C** | **2019** | 16 | 17 | 18 | 15 | 12 | 14 | 13 | 10 | 17 | 18 | 18 | 23 | 8.1 | 0.704 |
|  | **2020** | 25 | 8 | 15 | 17 | 13 | 11 | 11 | 13 | 18 | 9 | 14 | 16 | 16.4 | 0.128 |
|  | **2021** | 12 | 13 | 16 | 20 | 12 | 16 | 18 | 13 | 15 | 11 | 18 | 11 | 6.9 | 0.805 |
| **D** | **2019** | 647 | 515 | 559 | 606 | 621 | 547 | 628 | 598 | 491 | 526 | 461 | 651 | 80.3 | <0.001 |
|  | **2020** | 594 | 196 | 347 | 352 | 314 | 409 | 431 | 480 | 419 | 427 | 492 | 491 | 288.4 | <0.001 |
|  | **2021** | 459 | 445 | 457 | 511 | 531 | 450 | 603 | 604 | 511 | 425 | 558 | 537 | 84.2 | <0.001 |
| **E** | **2019** | 74 | 56 | 76 | 68 | 80 | 64 | 72 | 65 | 85 | 67 | 52 | 61 | 15.0 | 0.181 |
|  | **2020** | 61 | 19 | 39 | 36 | 46 | 41 | 46 | 55 | 49 | 43 | 59 | 62 | 35.8 | <0.001 |
|  | **2021** | 51 | 53 | 56 | 37 | 58 | 45 | 66 | 47 | 53 | 38 | 50 | 47 | 14.6 | 0.200 |
| **F** | **2019** | 74 | 56 | 76 | 68 | 80 | 64 | 72 | 65 | 85 | 67 | 52 | 61 | 15.0 | 0.181 |
|  | **2020** | 24 | 10 | 14 | 18 | 14 | 13 | 10 | 22 | 16 | 67 | 89 | 78 | 294.4 | <0.001 |
|  | **2021** | 67 | 23 | 26 | 32 | 52 | 33 | 50 | 31 | 25 | 23 | 25 | 29 | 62.8 | <0.001 |
