# Supplementary figures and images for: Seroprevalence of Epstein–Barr virus infection in children during the COVID-19 pandemic in Zhejiang, China
Source: Front Pediatr. 2023 Feb 9;11:1064330. doi: 10.3389/fped.2023.1064330 (PMC9947643; doi:10.3389/fped.2023.1064330)

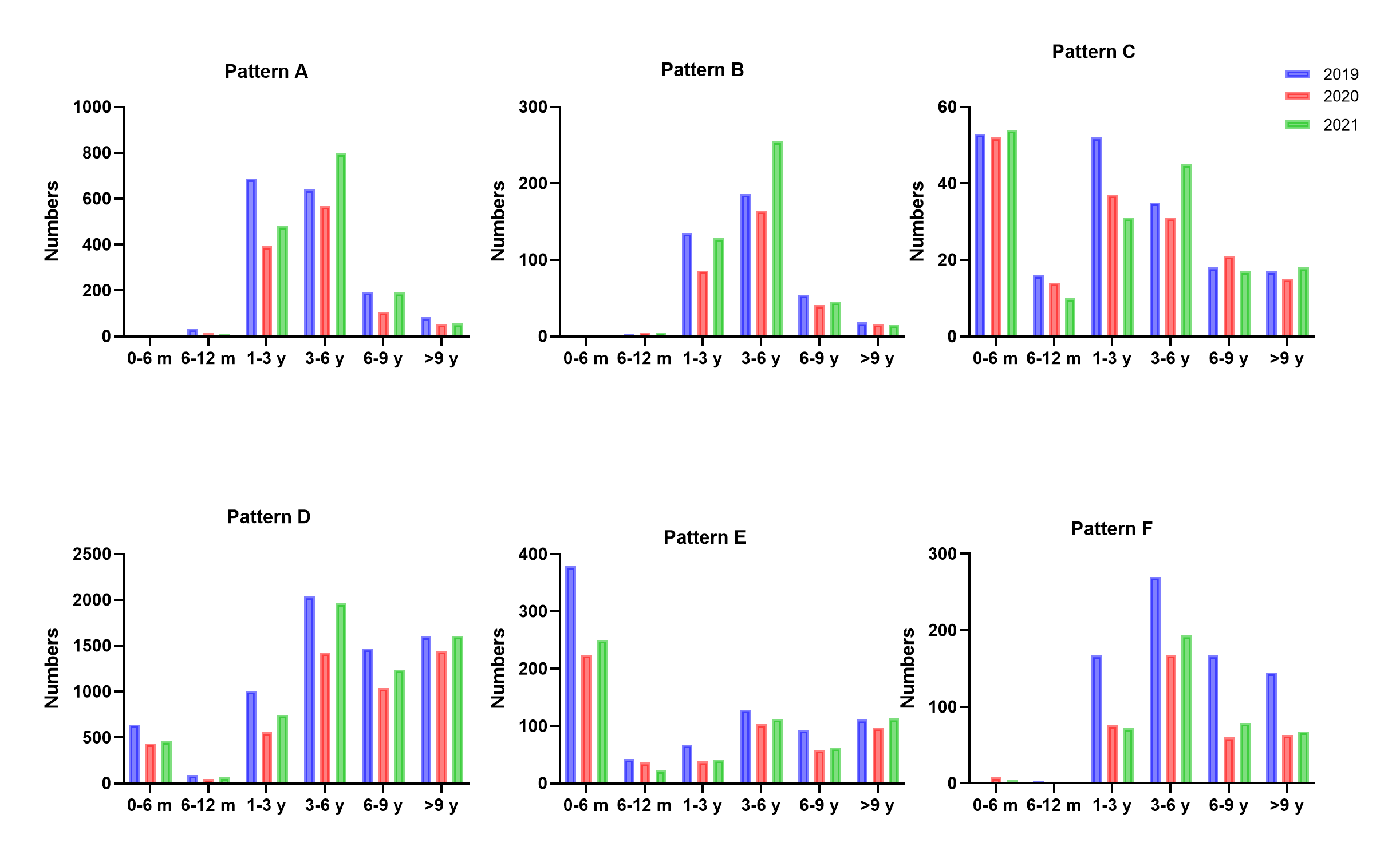

Supplement: Supplement Figure S1 — The number of EBV antibody patterns based on age between January 2019 and December 2021. [file Image1.tif]
